# Supplementary material for: Association of red cell distribution width and its changes with the 30-day mortality in patients with acute respiratory failure: An analysis of MIMIC-IV database
Source: PLoS One. 2023 Nov 3;18(11):e0293986. doi: 10.1371/journal.pone.0293986 (PMC10624277; doi:10.1371/journal.pone.0293986)
Supplement: S1 Table — (DOCX) [file pone.0293986.s001.docx]

**Table S1. Characteristics of AFR patients before and after the missing data interpolation**

| Variables | After interpolation (n=7497) | Before interpolation (n=7497) | Statistics | *P* |
| --- | --- | --- | --- | --- |
| BUN, M (Q_1_, Q_3_) | 23.00 (15.00, 40.00) | 23.00 (15.00, 40.00) | Z=0.014 | 0.989 |
| SpO_2_, Mean ± SD | 96.74 ± 3.63 | 96.76 ± 3.66 | t=-0.26 | 0.795 |
| Lactate, M (Q_1_, Q_3_) | 1.79 (1.30, 2.60) | 1.80 (1.20, 2.90) | Z=1.286 | 0.198 |
| Glucose, M (Q_1_, Q_3_) | 136.00 (108.00, 179.00) | 136.00 (108.00, 179.00) | Z=-0.000 | 1.000 |
| Bicarbonate, Mean ± SD | 22.33 ± 5.51 | 22.33 ± 5.51 | t=0.02 | 0.984 |
| Heart rate, Mean ± SD | 93.33 ± 21.30 | 93.29 ± 21.35 | t=0.10 | 0.919 |
| Weight, Mean ± SD | 82.07 ± 20.72 | 82.03 ± 22.67 | t=0.11 | 0.913 |
| INR, M (Q_1_, Q_3_) | 1.30 (1.20, 1.60) | 1.30 (1.10, 1.70) | Z=1.270 | 0.204 |
| Chloride, Mean ± SD | 103.55 ± 7.21 | 103.55 ± 7.21 | t=0.00 | 0.997 |
| PT, M (Q_1_, Q_3_) | 14.30 (12.80, 17.50) | 14.40 (12.70, 18.20) | Z=0.339 | 0.734 |
| Diastolic, Mean ± SD | 68.78 ± 17.65 | 68.62 ± 17.68 | t=0.53 | 0.593 |
| Temperature, Mean ± SD | 36.82 ± 0.81 | 36.83 ± 0.82 | t=-1.14 | 0.252 |
| Cr, M (Q_1_, Q_3_) | 1.10 (0.80, 1.80) | 1.10 (0.80, 1.80) | Z=-0.031 | 0.975 |
| FIO_2_, Mean ± SD | 67.40 ± 23.31 | 67.57 ± 25.07 | t=-0.40 | 0.687 |
| PTT, Mean ± SD | 39.65 ± 23.78 | 39.58 ± 24.99 | t=0.17 | 0.864 |
| SBP, Mean ± SD | 123.26 ± 24.60 | 123.15 ± 24.63 | t=0.26 | 0.791 |
| RR, Mean ± SD | 20.75 ± 5.97 | 20.77 ± 6.13 | t=-0.20 | 0.841 |
| WBC, M (Q_1_, Q_3_) | 11.80 (8.20, 16.60) | 11.80 (8.20, 16.60) | Z=-0.013 | 0.990 |

Z: rank sum test, t: t test

ARF: acute respiratory failure, BUN: blood urea nitrogen, M: median, Q_1_: 1st quartile, Q_3_: 3rd quartile, INR: international normalized ratio, PT: prothrombin time, Cr: creatinine, PTT: partial thromboplastin time, SBP: systolic blood pressure, RR: respiratory rate, WBC: white blood cell.
