# Supplementary material for: Association of red cell distribution width and its changes with the 30-day mortality in patients with acute respiratory failure: An analysis of MIMIC-IV database
Source: PLoS One. 2023 Nov 3;18(11):e0293986. doi: 10.1371/journal.pone.0293986 (PMC10624277; doi:10.1371/journal.pone.0293986)
Supplement: Attachment — Submitted filename: Response to reviewers comments.docx [file pone.0293986.s003.docx]

**Responses to Reviewers**

Dear Editor and reviewers,

We highly appreciate and thank the Editors and the reviewers for your time and valuable comments on this manuscript. We have carefully studied the comments and suggestions and revised our paper accordingly. The following are our point-by-point responses to the comments. We hope that the revisions are acceptable and that our responses adequately address the comments. Thank you for your consideration.

**Responses to Reviewer #1’s comments**

It's my great honor to have the opportunity to review the manuscript by ：Yuyi Shi et al. Through retrospective study with MIMIC-IV database, they found that high baseline RDW level and its changes during hospitalization was related to the increased risk of 30-day mortality in ARF and RDW changes may be a potential predictor for AKI short-term mortality. The manuscript is interesting and is somehow valuable for the effective management ARF. But there are some concerns need to be explained.

1. RDW T0 was divided into <14.9% and ≥14.9%. What is the basis for the grouping.

Response: Thank you for your comment. The RDW T0 was divided into <14.9% level and ≥14.9% level according to the median. Similarly, a previous study based on the MIMIC database that studied RDW was also divided it into low level and high level according to the median. We have added the description for the basis of the grouping in the manuscript.

Mo M, Huang Z, Huo D, Pan L, Xia N, Liao Y, Yang Z. Influence of Red Blood Cell Distribution Width on All-Cause Death in Critical Diabetic Patients with Acute Kidney Injury. Diabetes Metab Syndr Obes. 2022 Aug 2; 15:2301-2309. doi: 10.2147/DMSO.S377650.

2. I noticed there is a conceptual error in the sentence “the predictive value of SOFA combined RDW changes on 30-day mortality was superior to that of SOFA only with AUCs of 1.624 vs. 0.620”, in which on of the AUC arrived at 1.642 (more than 1). How to explain?

Response: Thank you for your comment. We are so sorry about this clerical error. The actual AUC value was 0.624, and we have revised it.

3. There are some mistakes in manuscript, such as “AKI” line 46 in page 3 should be “FRF”; “ARF one the common diseases -----” in line 50 of page 4 should be “ARF is one of the ------”.

Response: Thank you for your comment. We have changed “AKI” into “ARF”, “ARF one the common diseases -----” into “ARF is one of the ------”

4. The English needs to be improved.

Response: Thank you for your comment. We have carefully checked the manuscript, and revised the mistakes to improve our English writing. Also, we invited a native English speaker to help us revised this article.

**Responses to Reviewer #2’s comments**

Dear Prof. Vipa Thanachartwet,

This study was conducted as a retrospective cohort study by extract from the Medical Information Mart for Intensive Care IV (MIMIC-IV) database with the aim to explore the association between RDW and its changes and the 30-day mortality in ARF patients. This study concluded that high baseline RDW level and its changes during hospitalization was relate to the increased risk of 30-day mortality in ARF.

However, there are some comments for this manuscript to address as follows:

1. For the ethical concern in the study, this study was conducted as a retrospective cohort study and data were extracted from the Medical Information Mart for Intensive Care IV (MIMIC-IV) Database. The MIMIC was approved by the Institutional Review Boards (IRBs) of BIDMC and the MIT. No ethical approval of our IRB was required since this survey data were publicly available. The question is how to protect privacy information of included patients. This study is an observational study; therefore, the authors should state that this study was performed in accordance with the STrengthening the Reporting of OBservational studies in Epidemiology (STROBE) reporting guideline.

Response: Thank you for your comment. The MIMIC-IV database was publicly available and was approved by the IRBs of BIDMC and the MIT. How the MIMIC-IV to protect privacy information of included patients may refer to the relevant website description: https://www.physionet.org/content/mimiciv/2.2/. We also revised the ethical statement to clarify this study was performed in accordance with the STROBE reporting guideline.

2. Study participants, inclusion criteria and exclusion criteria should be clearly defined in Methods.

Response: Thank you for your comment. We have revised the Methods section to clearly define the study participants, inclusion criteria and exclusion criteria.

3. For variable collection, there are a lot of parameters included in the analysis. Some parameters such as SBP, DBP and MAP were related as well as BUN and Cr or etc. The related parameters may cause multicollinearity in the Cox regression analysis.

Response: Thank you and we are agree with your comment. We have performed the multicollinearity test on variables including age, ventilation time, temperature, respiratory rate, weight, FIO_2_, BUN, lactate, creatinine, chloride, PT, PTT, and CCI. The results were showed in the following table, and all the variance inflation factors (VIFs) all approached 1.

| Variables | VIF |
| --- | --- |
| age | 1.000293026 |
| ventilation time | 1.00000669 |
| temperature | 1.040151796 |
| respiratory rate | 1.000236109 |
| weight | 1.000050414 |
| FIO_2_ | 1.00000495 |
| BUN | 1.000059585 |
| lactate | 1.002710493 |
| creatinine | 1.032349135 |
| chloride | 1.000057775 |
| PT | 1.000030777 |
| PTT | 1.000004819 |
| CCI | 1.011766789 |

4. In outcomes and follow-up, the study outcome was 30-day mortality. The MIMIC-III followed up by information in the electronic medical charts and hospital department records, or making contact with the patients, their family members, their attending health care workers, or family physicians on the phone. So, this study was extracted from MIMIC-IV or MIMIC-III Database. How to protect confidentiality of the study participants as “making contact with the patients, their family members, their attending health care workers, or family physicians on the phone”?

Response: Thank you and we are agree with your comment. The MIMIC-IV aims to carry on the success of MIMIC-III, which adopted a permissive access scheme which allowed for broad reuse of the data. The protection for the confidentiality of the study participants run through the creation of MIMIC-IV carried out steps. Patient identifiers as stipulated by HIPAA were removed. Patient identifiers were replaced using a random cipher, resulting in deidentified integer identifiers for patients, hospitalizations, and ICU stays. Structured data were filtered using look up tables and allow lists.

1. Johnson, A. E., Pollard, T. J., Shen, L., Lehman, L.H., Feng, M., Ghassemi, M., ... & Mark, R. G. (2016). MIMIC-III, a freely accessible critical care database. Scientific data, 3(1), 1-9.

5. In statistical analysis,

a. On page 7, “chi-square test was used for cooperation”, what is the meaning of cooperation?

Response: Thank you for your comment. We are so sorry for the clerical error, and we have revised the “cooperation” into “comparation.”

b. On page 8, “Univariable Cox regression and bidirectional stepwise regression analyses were used to screen the covariates related to the 30-day mortality. Univariable and multivariable Cox regression models were established to explore the relationship of RDW T0, RDW T1, and RDW changes and the 30-day mortality in ARF patients with hazard ratios (HRs) and 95% confidence intervals (CIs).” This is rather confusing which method of multivariate analysis was performed in this study.

Response: Thank you for your comment. We have revised the description for the methods of statistical analyses.

c. On page 8, “the RDW T0 levels were divided into RDW T0 <14.9% and RDW T0 ≥14.9%.” How and why this cutoff value was selected?

Response: Thank you for your comment. The RDW T0 was divided into <14.9% level and ≥14.9% level according to the median. Similarly, a previous study based on the MIMIC database that studied RDW was also divided it into low level and high level according to the median. We have added the description for the basis of the grouping in the manuscript.

Mo M, Huang Z, Huo D, Pan L, Xia N, Liao Y, Yang Z. Influence of Red Blood Cell Distribution Width on All-Cause Death in Critical Diabetic Patients with Acute Kidney Injury. Diabetes Metab Syndr Obes. 2022 Aug 2; 15:2301-2309. doi: 10.2147/DMSO.S377650.

d. On page 8, “We also drew receiver operator characteristic curves (ROCs) with areas under curve (AUCs) to assess the predictive performance of RDW changes combined with SOFA on the 30-day mortality of ARF.” Why RDW changes were combined with SOFA in the analysis?

Response: Thank you for your comment. The SOFA score is a validated prognostic score ranging from 0–24, with points assigned for evidence of organ failure within 6 different organ systems, with higher scores correlating with a higher likelihood of in-hospital mortality. This study aimed to explore the association between RDW and its changes and the 30-day mortality in ARF. Also, we assessed the predictive performance of RDW changes on 30-day mortality in ARF, using the SOFA as a comparison object. Xu et al. developed and validated a nomogram to predict the mortality risk in elderly patients with ARF, in which they similarly compared the predictive performance of their nomogram with SOFA score.

Ferreira FL, Bota DP, Bross A, Mélot C, Vincent J-L. Serial evaluation of the SOFA score to predict outcome in critically ill patients. JAMA. 2001;286(14):1754–8. doi: 10.1001/jama.286.14.1754

Raith EP, Udy AA, Bailey M, McGloughlin S, MacIsaac C, Bellomo R, et al. Prognostic accuracy of the SOFA score, SIRS criteria, and qSOFA score for in-hospital mortality among adults with suspected infection admitted to the intensive care unit. JAMA. 2017;317(3):290–300. doi: 10.1001/jama.2016.20328

Xu J, Weng J, Yang J, Shi X, Hou R, Zhou X, Zhou Z, Wang Z, Chen C. Development and validation of a nomogram to predict the mortality risk in elderly patients with ARF. PeerJ. 2021 Mar 9;9:e11016. doi: 10.7717/peerj.11016. PMID: 33854838; PMCID: PMC7953875.

6. In the results,

a. On page 9, “4,188 (55.86%) of them were females” but “4188 (55.86)” were male in the Table 1.

Response: Thank you for your comment. We are so sorry for the clerical error, and we have revised the “females” into “males.”

b. Please revised this sentence “The average RDW T0 between survival and 30-day mortality groups were 15.22% vs. 16.20%, and that of RDW T1 were 15.41% vs. 16.46%.” on page 9 as this may make misinterpreted to the readers.

Response: We have revised this sentence.

c. On page 12, what is the meaning of “first care unit”?

Response: Thank you for your comment. The variable “first care unit” mean the type of the ICU when the patients hospitalized in at the first time, including MICU, SICU and others. To make it clear, we have changed the “first care unit” into “type of ICU at first hospitalization”.

d. On page 12, what is the meaning of “significantly linked” and “linked” in the first and second paragraph?

Response: Thank you for your comment. We have revised the “linked to” into “related to.”

e. On page 16, please check “AUCs of 1.624”.

Response: Thank you for your comment. We are so sorry about this clerical error. The actual AUC value was 0.624, and we have revised it.

7. In Table 1,

a. Is it possible to collect all variables of 7,497 patients without any missing data?

Response: Thank you for your comment. The study data were extracted from the MIMIC-IV database, it was not realistic to collect all variables of 7,497 patients without any missing data. However, the missing data which proportion over 20% were deleted, otherwise were interpolated by the random forest method. In addition, we performed the sensitivity analysis of the participants’ characteristics before and after the interpolation, and the results showed that there was no significant difference them.

b. It is wonder why the “RDW T0 and T1” are expressed as %, Mean ± SD, but the “RDW changes” are expressed as %, M (Q1, Q3).

Response: Thank you for your comment. We have mentioned in the Statistical analysis section that normal distributed data were described by Mean ± SD, while skewed distribution data were described by M (Q1, Q3). The RDW T0 and T1 were normal distributed data, and the RDW change was skewed distribution data.

c. It is wonder why the “RDW T0 (<14.9% vs. ≥14.9%) and RDW T1 (<15.1% vs. ≥15.1%)” have different cutoff values.

Response: Thank you for your comment. We divided the RDW T0 and T1 into two levels respectively according to their own median. The median of RDW T0 among participants was 14.9%, while the median of RDW T1 was 15.1%.

d. Please define “LOS” in the footnote of Table 1.

Response: Thank you for your comment. We have added the definition of “LOS” in the footnote.

e. The characteristics and baseline data of ARF patients were compared between survival and 30-day mortality group to determine the associated factors for 30-day mortality. It is wonder why all variables were included in the univariate Cox regression model in Table 2.

Response: Thank you for your comment. Table 2 showed the process of covariates screening. We used univariable Cox regression analysis and bidirectional stepwise regression analysis to screening the covariates related to 30-day mortality in ARF. All the variables were included in the univariable Cox regression analysis, and those with P values <0.05 were considered the potential covariates that were then included into the bidirectional stepwise regression analysis. Finally, variables with P values <0.05 in the bidirectional stepwise regression analysis were the covariates for further adjustment.

8. In Table 2,

a. Why MICU was choose as reference in first care unit?

Response: Thank you for your comment. Table 2 showed the process of covariates screening. When take classified variables into consideration, we picked one of the categories at random as the reference. In other words, it is not obligatory to use the “MICU” as a reference, we can also choose “SICU” or “others” as the reference.

b. Why this “Charlson comorbidity index” was used?

Response: Thank you for your comment. The Charlson comorbidity index (CCI) reflects the severity of illness, which may influence the 30-day mortality in patients with ARF. We included it into the covariates screening, and found it was significantly associated with the 30-day mortality. In turn, when we explored the association between RDW and its changes and the 30-day mortality in patients with ARF, we adjusted for the CCI.

Tuty Kuswardhani RA, Henrina J, Pranata R, Anthonius Lim M, Lawrensia S, Suastika K. Charlson comorbidity index and a composite of poor outcomes in COVID-19 patients: A systematic review and meta-analysis. Diabetes Metab Syndr. 2020 Nov-Dec;14(6):2103-2109. doi: 10.1016/j.dsx.2020.10.022.

9. Table 4 is unclear.

Response: Thank you for your comment. We have revised the Table 4.

10. In Table 5, how were “SOFA + RDW changes” combined in the analysis?

Response: Thank you for your comment. We compared the predictive performance of SOFA combined with RDW changes with that of only SOFA score on 30-day mortality in patients with ARF. The “SOFA + RDW changes” was regarded as a multivariable model which was composed of variables including “SOFA” and “RDW changes”. We aimed to explore whether the predictive value of SOFA score combined with RDW changes on the 30-day mortality in patients with ARF is better than that of single SOFA score, in order to assess the prognostic value and complementary significance of RDW to the existing scoring system in clinical.

11. In Table 6,

a. How to determine the cutoff values?

Response: Thank you for your comment. The cutoff value was calculated using the Youden index, which was a summary measure widely used in the evaluation of the diagnostic accuracy of a medical test.

Habibzadeh F, Habibzadeh P, Yadollahie M. On determining the most appropriate test cut-off value: the case of tests with continuous results. Biochem Med (Zagreb). 2016 Oct 15;26(3):297-307. doi: 10.11613/BM.2016.034.

b. “The predictive value of SOFA combined RDW changes on 30-day mortality was superior to that of SOFA only with AUCs of 1.624 vs. 0.620.” on page 16, but the accuracy of SOFA + RDW changes was only 0.581 (0.566-0.597) as shown in Table 6. Please concern using “superior” in this sentence.

Response: Thank you for your comment. We have revised the “superior to” into “a little better than.”

12. Table S1 is not mention in the result.

These are all issues raised for this manuscript and major revision is needed.

Response: Thank you for your comment. We have added the Table S1 in the result section.
