# Supplementary material for: Association of red cell distribution width and its changes with the 30-day mortality in patients with acute respiratory failure: An analysis of MIMIC-IV database
Source: PLoS One. 2023 Nov 3;18(11):e0293986. doi: 10.1371/journal.pone.0293986 (PMC10624277; doi:10.1371/journal.pone.0293986)
Supplement: Attachment — Submitted filename: Response to reviewers comments.docx [file pone.0293986.s004.docx]

**Responses to Reviewers**

Dear Editor and reviewers,

We highly appreciate and thank the Editors and the reviewers for your time and valuable comments on this manuscript. We have carefully studied the comments and suggestions and revised our paper accordingly. The following are our point-by-point responses to the comments. We hope that the revisions are acceptable and that our responses adequately address the comments. Thank you for your consideration.

**Responses to Reviewer #1’s comments**

In table 3, 4, it included “RDW changes <0%”. What does it mean by RDW changes <0%? The minimal rate of change for any indicator is zero and should not be less than zero. Please explain.

Response: Thank you for your comment. In this study, RDW change values were not the absolute value. The RDW changes were calculated by RDW T1 - RDW T0, and when RDW level at T0 higher than that at T1, the value of RDW change was <0%.

**Responses to Reviewer #2’s comments**

The revised manuscript is much improved. However, there are minor comments for this manuscript to address as follows:

1. In outcomes and follow-up (page 7, line 125), in order to protect confidentiality of the study participants, method of deidentification of study participants in the responses to the reviewers should be added in this section.

Response: Thank you for your valuable comment. We have added the description of deidentification of study participants in this section.

2. In page 8, line 149, how important of SOFA score should be added from response to the reviewers.

Response: Thank you for your valuable comment. We have added the importance of SOFA score from the response to the reviewers.

3. Table S1 is disappeared.

Response: Thank you for your comment. We are very sorry for the mistake, and we have re-uploaded the Table S1.

4. In the results (page 9, line 169-170), this is only descriptive, but no interpretation of results.

Response: Thank you for your valuable comment. We have revised this paragraph into description for the interpretation of results. Table 1 showed the characteristics of participants, and the comparation between groups was base on the crude model, which we think could not indicate the real association between the baseline variables and the study outcome.

5. Table 1 and Table 3, RDW T0, RDW T1, and RDW changes was classified into categorical variables, but these are not mention in the results. The categorical data of RDW changes was overlapping (<0%, 0-0.3%, ≥0.3%). If the data was 0.3%, how to classify.

Response: Thank you for your valuable comment. We have added the description of RDW T0, RDW T1, and RDW changes as categorical variables in the methods section. Also, the intervals of RDW changes have been revised into <0%, 0%-0.29%, and ≥0.3%.

6. In the results (page 13, line 199), “High RDW T0” did not mention before, therefore it is difficult to follow.

Response: Thank you for your valuable comment. We have revised this sentence to make it clear.

7. Table 2-6, there was no “n” number of sample for each variable.

Response: Thank you for your comment. The number of samples for each variable has been added in these tables.

8. The description of results for Table 4 (page 15, line 220-222) was unclear.

Response: Thank you for your valuable comment. We have revised the description of results for Table 4.

9. Table 6, How to determine the cutoff values?

Response: Thank you for your comment. The cutoff value was calculated by the Youden index, which reflects the optimum cutoff of the prediction probability. More details of the ROC curve and Youden index could reference the following article. Also, in our study, we mainly used the AUC, sensitivity, and specificity of ROC curves to assess the predictive performance of RDW changes on 30-day mortality in ARF.

Bantis LE, Tsimikas JV, Chambers GR, Capello M, Hanash S, Feng Z. The length of the receiver operating characteristic curve and the two cutoff Youden index within a robust framework for discovery, evaluation, and cutoff estimation in biomarker studies involving improper receiver operating characteristic curves. Stat Med. 2021 Mar 30;40(7):1767-1789. doi: 10.1002/sim.8869.

10. In discussion (page 21, line 309-310), it is wonder that the study population were patients with ARF, how to compare AKI and high WBC with those without ARF.

These are all issues raised for this manuscript and minor revision is needed.

Response: Thank you for your comment. We are very sorry for this clerical error. The comparation has been between survival group and 30-day mortality group in this study. We have revised this sentence into “In our study population, 92.10% ARF patients who died in the hospital had AKI and a higher WBC count compared with the survivors.”
